# A Plate Based Assay for Determination of the Median Lethal Dose of 1-Hydroxyphenazine in *Caenorhabditis elegans*

**DOI:** 10.17912/micropub.biology.000352

**Published:** 2021-01-13

**Authors:** Muhammad Zaka Asif, Victoria L. Van der Gaag, Jane Guo, Kelsey A. Nocilla, Cole J. Muzio, Arthur S. Edison

**Affiliations:** 1 Department of Biochemistry & Molecular Biology and Complex Carbohydrate Research Center, University of Georgia; 2 Department of Genetics; 3 Institute of Bioinformatics

## Abstract

*Caenorhabditis* *elegans* is an ideal model organism for studying the xenobiotic detoxification pathways of various natural and synthetic toxins. We developed a new workflow to study the effects of 1-hydroxyphenazine (1-HP), a toxin produced by the bacterium *Pseudomonas aeruginosa*, on the survival of *C*. *elegans*. Prior research has demonstrated that *C. elegans* can detoxify 1-HP through the general mechanism of O-glycosylation. As part of the Vertically Integrated Projects (VIP) undergraduate research team, we have developed a workflow for a plate-based toxicity assay to determine the median lethal dose (LD50) of 1-HP. This was achieved through a toxin exposure method in which the worms were exposed to varying concentrations of 1-HP. The death rates were measured using a fluorescent bead assay. This workflow can be used to test *C*. *elegans* responses to different toxins and also the response of different mutant strains to a toxin of interest.

**Figure 1. Determining the Median Lethal Dose for 1-Hydroxyphenazine in C. elegans:**
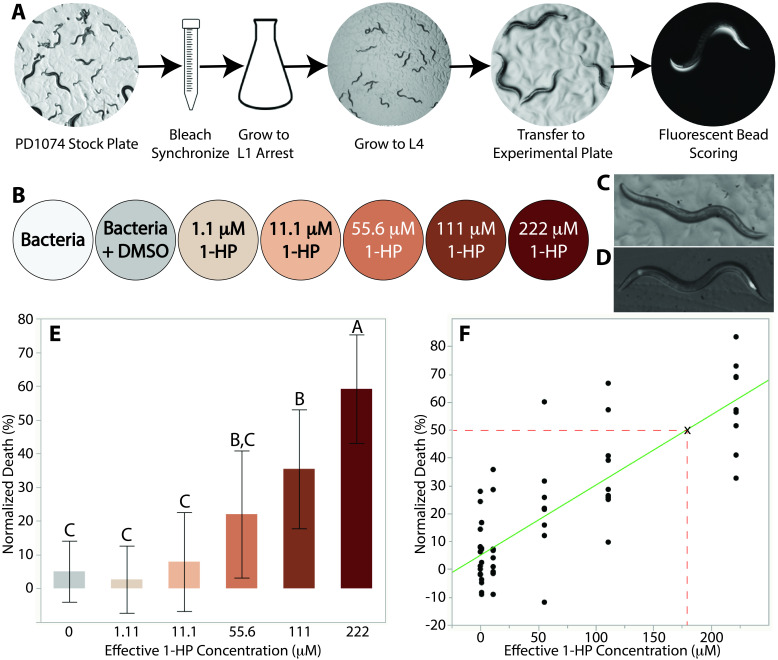
**A)** A stock plate of PD1074 worms at varying life stages was washed with M9 and transferred to a 15 mL conical tube. The worms were bleach synchronized and the eggs were resuspended and left to grow in M9 for 24 hours. The L1 arrested worms were transferred to 10 cm plates and left to grow to L4 for 35-42 hours. Approximately 15 L4 worms were transferred onto prepared experimental plates and incubated for 7 hours. Using a fluorescent bead assay, the worms were scored as dead or alive. **B)** Each replicate consisted of 7 plates, 2 controls and 5 toxin plates of varying concentrations. The applied concentrations of 1-HP were 90 x greater than the effective concentrations, which were corrected by the volume of the agar. **C)** A gravid adult PD1074 worm without exposure to fluorescent beads. **D)** A gravid adult worm following 5 min exposure to fluorescent beads. **E)** Mean normalized death (y-axis) plotted against effective 1-HP concentration (x-axis). Data labeled by the same letter (A, B, or C) are not significantly different, while those labeled by different letters are statistically different with a p-value < 0.05. **F)** A linear fit curve applied to the mean normalized death vs. effective 1-HP concentration yielded the following equation: y=0.25x+5.23. Using this equation, the effective LD50 concentration is 179 μM.

## Description

1-Hydroxyphenazine (1-HP) is a small molecule produced by *Pseudomonas aeruginosa,* a bacterium that is used for pathogenesis models in *C.* *elegans* (Cezairliyan *et al.*, 2013; Mahajan-Miklos, Tan, Rahme, & Ausubel, 1999). 1-HP is an especially interesting toxin to study as it has been shown to interact with human cells causing ciliary-slowing associated with dyskinesia and ciliostasis (Wilson *et al.*, 1987). Prior research in our lab has shown that this molecule is toxic to *C. elegans*, with an LD50 between 150 and 200 μM, but *C.* *elegans* can glycosylate 1-HP, which detoxifies the molecule (Stupp *et al.*, 2013).

We have developed a modified, plate-based assay to determine the LD50 in the PD1074 strain of *C.* *elegans.* PD1074 is the recommended strain for genomics and genetic experiments that will utilize the new reference genome VC2010 (Yoshimura *et al.*, 2019). Importantly, PD1074 minimizes ambiguity caused by highly divergent N2 strains (Gems & Riddle, 2000; Sterken, Snoek, Kammenga, & Andersen, 2015; Vergara *et al.*, 2009).

To ensure that our results were not affected by differences in life stage, a mixed-stage population of PD1074 was synchronized and then allowed to grow to L1 arrest (Porta-de-la-Riva, Fontrodona, Villanueva, & Ceron, 2012) (Figure 1A). The worms remained in M9 media for over 24 hours to ensure that eggs had hatched and then were transferred to 10 cm plates with an OP50 *E. coli* lawn and allowed to grow for 36-39 hours until they reached the L4 stage (Figure 1A). The worms were then washed off the plates with M9 and an average of 15 worms were pipetted onto 6 cm plates. All control and experimental plates in a replicate were seeded by the same synchronized batch. Each of these plates corresponded to one of five toxin concentrations or one of two controls and the worms were left to incubate for 7 hours (Figure1B). This time optimized the number of worms killed while avoiding developmental progression to young adult. Our toxin concentration range was derived from prior work done on N2 (Stupp *et al.*, 2013).

After incubation with the toxin, we used a fluorescent bead solution to visualize the worms under a fluorescent microscope (Kiyama, Miyahara, & Ohshima, 2012; Nika, Gibson, Konkus, & Karp, 2016). This was done in order to determine the number of worms that died during the incubation period. Worms with any observed fluorescence in their pharynx and anus were counted as alive while those that did not fluoresce were counted as dead (Figure 1C/D). Fluorescence, instead of movement, was used as a marker for alive worms as the effects of 1-HP on *C. elegans* motility are still not fully understood, so it is possible that the toxin could cause partial or complete paralysis. Indeed, we observed very little movement amongst worms exposed to relatively high (>55.6 μM) concentrations of 1-HP*.*

Our data show that at 1-HP experimental concentrations of 222 μM, 111 μM, and 55.6 μM, mortality rates are approximately 60%, 40%, and 20%, respectively. The difference of mortality rates between the control and lower concentrations were statistically insignificant (Figure 1E). We performed a Tukey’s honestly significant difference (HSD) test using JMP statistical software that showed that the death at 222 μM was statistically significant compared to all other toxin concentrations in our range (p<0.0001) (Figure 1E). We also found that the death rate at 111 μM was statistically significant when compared to the death rates at the other toxin concentrations and controls except for 55.6 μM (p<0.003) (Figure1E). The data show that 1-HP is toxic to PD1074 at similar concentration ranges on plates as it was to N2 in liquid (Stupp *et al.*, 2013). Furthermore, by providing results which correspond with prior literature, we have shown that our plate-based assay is a reliable workflow for LD50 determination. We performed a regression analysis on our data in order to calculate the LD50 for PD1074 and found the LD50 to be 179 μM (Figure 1F). This is consistent with the value reported for N2 (between 150 and 200 μM) (Stupp *et al.*, 2013). This suggests that 1-HP has a similar toxicity to PD1074 as it does to N2, which is to be expected due to the genetic similarities between the two strains.

This study not only provides evidence that PD1074 has a similar response to 1-HP as N2, it also provides a novel method for a plate-based assay to determine the LD50 in *C. elegans.* Using this technique, in the future we will screen mutants in order to help determine which genes are associated with 1-HP glycosylation.

## Methods

***Strain, Growth, and Media*:** The PD1074 strain of *C. elegans* was grown and maintained on 10 cm NGM agar plates seeded with an LB-cultured, OP50 strain of *Escherichia coli* at 22° C. The worms were chunked onto new 10 cm seeded plates once a week. Additionally, a 10 cm stock plate of PD1074 *C. elegans* was stored at 15° C. Every three to four weeks, a new set of 10 cm plates were chunked from the stock plate in order to maintain a homogeneous experimental population and prevent significant genetic drift.

***Experimental Plate Preparation:***A stock concentration of 1-HP was diluted using DMSO in order to produce the following five experimental concentrations: 100 μM, 1 mM, 5 mM, 10 mM, and 20 mM. The concentrations at plate surface are the effective concentrations and were corrected by the ratio of the volume of the agar to the volume of DMSO (90 x). 100 μL of each toxin concentration was distributed onto the total surface area of 6 cm NGM plates using a cell spreader and left to dry for 30 min. Then, each toxin plate was seeded with 25 μL of OP50 bacteria and spread to create a small lawn at the center of the plate. After an additional 30 minutes of drying, the plates were placed at 22°C in an insulated container overnight.

***Bleach Synchronization:*** A plate of *C. elegans* was washed with 3 mL of M9 and pipetted into a 15 mL falcon tube. A second wash of the plate was performed with an additional 3 mL of M9 and left to sit for 5 min to ensure removal of any remaining worms. After 5 min, the contents were pipetted into the falcon tube. The worm/M9 mixture was centrifuged for 1 min at 2000 rpm. A worm pellet was formed at the bottom of the tube and isolated through aspiration. The pellet was then mixed with 2.5 mL of the bleach solution (Porta-de-la-Riva *et al.*, 2012). The tube was shaken gently to disperse the pellet throughout the solution. Every 30 sec, 5 μL of the worm solution was aliquoted onto a glass slide and observed under a microscope to check the state of the worms. Once eggs could be seen breaking out of the worm bodies, the bleaching process was stopped by adding 12.5 mL of M9 and quickly inverting the tube to ensure dilution. The solution was centrifuged for 1 min at 2000 rpm and the supernatant was aspirated. The pellet underwent 2 additional washes and centrifugations at 1200 rpm for 3 min. The egg pellet was resuspended in 5 mL of M9 and then transferred to a 25 mL Erlenmeyer flask and covered with foil. The flask was left to shake at 300 rpm in a 20°C shaker for 24 hours until the worms reached L1 arrest life-stage.

***Experimental Worm Plating:*** After the worms reached the L1 stage, the worms were transferred to a 15 mL falcon tube. The liquid culture was centrifuged for 3 min at 1200 rpm and the supernatant was aspirated to leave a volume of about 300 μL in the tube. The tube was gently shaken to disperse the worm pellet and the 300 μL mixture was pipetted on the outer edge of a seeded 10 cm plate. The plate of L1 worms were incubated at 22° C for about 35-42 hours until they reached the L4 life-stage. Then the worms were washed with 3 mL M9 and pipetted into a 15 mL falcon tube, twice. An average of 15 L4 worms were aliquoted onto each experimental toxin plate. This was done by aliquoting 10 μL of the worm and M9 mixture onto a glass slide to determine the average amount of worms present per μL of M9. Based on this the volume of M9 to be aliquoted onto the experimental plate was determined. After the M9 dried, the experimental plates were placed in an incubator set at 22° C for an exposure time of 7 hours.

***Fluorescent Bead Assay and Data Collection:***A fluorescent bead assay was used to distinguish between living and dead worms following toxin exposure. The solution was prepared with Fluoresbrite® Polychromatic Red Microspheres 0.5µm, according to a preexisting protocol (Kiyama *et al.*, 2012). After the 7-hour exposure period, the bead solution was dropped in 2 μL aliquots onto each worm on the experimental plates. The worms were left to feed on the bead solution for 5 minutes, and then observed under a fluorescent microscope with a Texas Red Filter. If there was visible fluorescence in the pharynx or anus, the worms were scored as alive. If there was no visible fluorescence, then the worms were scored as dead. Each plate was scored twice, by separate researchers, and the counts were averaged to determine the final count.

***Statistical Analysis:***The statistical test performed was a Tukey-Kramer HSD test. This test has three assumptions: observations being tested are independent within and among the groups, the groups associated with each mean in the test are normally distributed, and there is homogeneity of variance (Montgomery, 2013). Our data met these three assumptions thus we chose to carry out this test. The Tukey HSD test was performed using a publicly available software (Pro, 2015).
